# Biomass dynamics in a logged forest: the role of wood density

**DOI:** 10.1007/s10265-018-1042-9

**Published:** 2018-05-30

**Authors:** Vu Thanh Nam, Niels P. R. Anten, Marijke van Kuijk

**Affiliations:** 1 Department of Biology, Utrecht University, Padualaan 8, 3584 CH Utrecht, The Netherlands; 2 Present Address: Vietnam Administration of Forestry, No 2, Ngoc Ha, Ba Dinh, Hanoi, Vietnam; 3 Centre for Crop Systems Analysis, Wageningen University, Droevendaalsesteeg 1, 6708 PB Wageningen, The Netherlands

**Keywords:** Biomass, Carbon dynamics, Demographic rates, Tropical forest, Vietnam

## Abstract

Wood density (WD) is believed to be a key trait in driving growth strategies of tropical forest species, and as it entails the amount of mass per volume of wood, it also tends to correlate with forest carbon stocks. Yet there is relatively little information on how interspecific variation in WD correlates with biomass dynamics at the species and population level. We determined changes in biomass in permanent plots in a logged forest in Vietnam from 2004 to 2012, a period representing the last 8 years of a 30 years logging cycle. We measured diameter at breast height (DBH) and estimated aboveground biomass (AGB) growth, mortality, and net AGB increment (the difference between AGB gains and losses through growth and mortality) per species at the individual and population (i.e. corrected for species abundance) level, and correlated these with WD. At the population level, mean net AGB increment rates were 6.47 Mg ha^−1^ year^−1^ resulting from a mean AGB growth of 8.30 Mg ha^−1^ year^−1^, AGB recruitment of 0.67 Mg ha^−1^ year^−1^ and AGB losses through mortality of 2.50 Mg ha^−1^ year^−1^. Across species there was a negative relationship between WD and mortality rate, WD and DBH growth rate, and a positive relationship between WD and tree standing biomass. Standing biomass in turn was positively related to AGB growth, and net AGB increment both at the individual and population level. Our findings support the view that high wood density species contribute more to total biomass and indirectly to biomass increment than low wood density species in tropical forests. Maintaining high wood density species thus has potential to increase biomass recovery and carbon sequestration after logging.

**Electronic supplementary material:**

The online version of this article (10.1007/s10265-018-1042-9) contains supplementary material, which is available to authorized users.

## Introduction

Tropical forests play a prominent role in driving the global carbon cycle (Malhi et al. 2011), yet the mechanisms driving carbon dynamics in tropical forests are still poorly understood. One of the major challenges in studying forest carbon dynamics involves the quantification of biomass and its relation to the structure and species composition of the forest. In tropical forest ecosystems, carbon is mainly stored in living biomass in standing trees and soil, while a smaller amount is stored in litter and dead wood (Malhi et al. 2009; Ngo et al. 2013; Sierra et al. 2007). Most studies focus on aboveground biomass (AGB) (Chave et al. 2008a, b; Lewis et al. 2013; Malhi et al. 2004; Vieira et al. 2004) where living tree biomass is typically estimated using allometric models (Chave et al. 2005).

Biomass dynamics in forests are driven by the amount of standing biomass on the one hand and the individual rates of growth, recruitment and mortality on the other (Chave et al. 2008a; Keeling et al. 2008). Amounts of standing biomass in mature tropical forests may differ considerably both within and between regions (Slik et al. 2010). Similarly, biomass dynamics through growth, recruitment and mortality also differ widely between different tropical forests (e.g. Chave et al. 2008a, b; Djomo et al. 2011; Malhi et al. 2004, 2009).

One of the factors that may affect the variation in biomass and associated biomass dynamics of forests is the species composition and associated distribution of functional traits (Iida et al. 2012; Poorter et al. 2008, 2010; Vieira et al. 2004). In this respect, wood density (WD) is often used as one of the key traits to indicate functional groups (Muller-Landau 2004). Low WD species are associated with rapid growth and a resource acquisitive growth strategy, while high WD species are associated with slow growth and stress resistance (Chave et al. 2005, 2009). As such, variation in demographic rates between species can be linked to WD (Hietz et al. 2013). Furthermore, as WD represents the amount of mass and carbon per unit volume, it is directly linked to forest carbon stocks. This raises the question to what extent variation in WD drives variation in tree demographic rates at the individual level and how this affects carbon dynamics at the population level.

A number of studies found a fairly consistent negative correlation between WD and mortality rates, suggesting that high WD species tend to live longer and could thus grow to greater size (King et al. 2006; Muller-Landau 2004; Wright et al. 2010). But results regarding growth are less clear. Low WD species were shown to exhibit faster rates of diameter growth than high WD ones (Muller-Landau 2004; Wright et al. 2010), as was predicted by theoretical models (Anten and Schieving 2010). But, as low WD species entails less mass per unit volume, it is unclear whether this faster diameter growth also translates into faster biomass increment. Unfortunately, few studies have considered relationships between WD and AGB increment rates. Contrary to the notion that high WD species are associated with slow growth, Keeling et al. (2008) found positive correlations between WD and AGB increment rates, at least on fertile sites in a mature tropical Amazonian forest.

In order to scale from species individual plant differences in demographic rates to population level biomass dynamics, differences in abundance between species should be taken into account. The relative abundance of species with different WD may vary depending on environmental conditions and disturbance history (Ketterings et al. 2001; Wiemann and Williamson 2002). To our knowledge however there is no study that has determined the relationship between variation in WD and biomass dynamics associated with growth, recruitment and mortality, both at the individual and population level. Such an analysis would strongly contribute to our understanding of the processes that play a role in forest carbon dynamics (Keeling et al. 2008). It may also contribute to forest management especially in tropical forests, where selective logging regimes are often implemented. Selective logging tends to focus on harvesting commercial species with often relatively high WD (Sist et al. 2014), and it is therefore important to determine how the associated changes in species composition could impact biomass dynamics of the forest (Bunker et al. 2005).

This study focuses on the biomass dynamics after logging in tropical forest stands, with particular emphasis on the role of interspecific variation in WD. We address two questions: First, how much biomass and carbon is stored in the forest and how is it distributed among different components? Second, to what extent are demographic rates and biomass dynamics associated with wood density, across species? These questions are addressed for a forest in the Central Highland of Vietnam, which was selectively logged 30 years prior to our study.

## Materials and methods

### Study site and plot layout

**Fig. 1:**
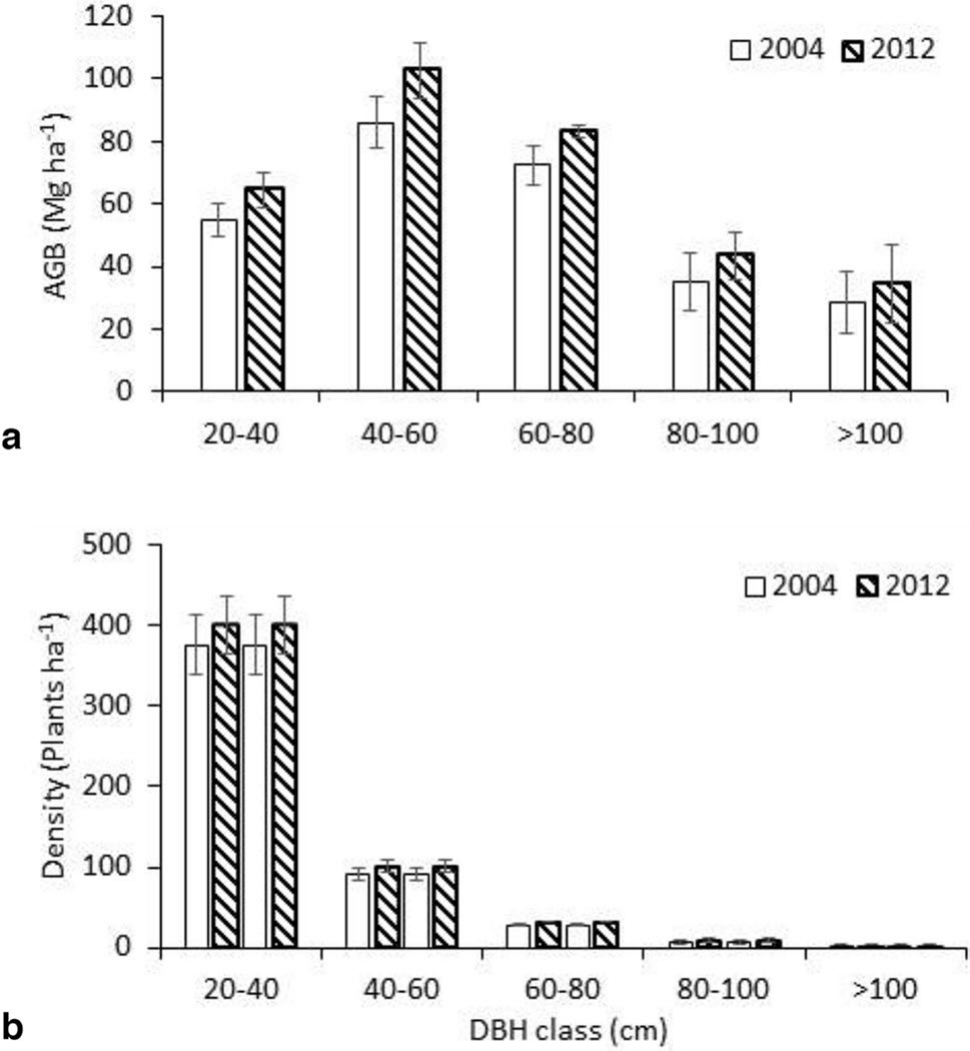
Dynamics of aboveground biomass (**a**) and tree density (**b**) in 2004 and 2012

The study was conducted in an evergreen forest (108°17′75″E and 14°35′35″N) in K’Bang district, Gia Lai province, in the Central Highland of Vietnam. The topography of the area is mostly flat with an altitude of 500–600 m. Annual precipitation is approximately 2,300 mm with a 3–4 months dry season. Mean annual air humidity is 82% and mean annual temperature is 23 °C (GSO 2013). The soils in the area are classified as Ferralsols (Lung et al. 2011). A map of the location of the study site is provided in the supporting information (see Fig. 1, S1).

The forest at the study site was selectively logged for the first time between 1980 and 1982 with a harvesting intensity of about 30–35% of the standing volume and focusing solely on species producing timber suitable for construction. A total of six permanent plots (100 m × 100 m) were established and inventoried in the study site in 2004 by the Highland Tropical Forest Research Centre (hereafter Highland FRC). The forest was never logged again, therefore the plots had a 30–32 years recovery period in 2012. We collected additional data in the same six permanent plots in 2012.

### Measurements on aboveground components

At each inventory (in 2004 and 2012) in each permanent plot, all trees with a diameter at breast height (DBH) larger than or equal to 10 cm were identified at the species level based on Vietnamese guidelines (MARD 2000) and numbered. Across these six plots, a total of 105 species were found. Of each individual, DBH (using a diameter tape accuracy of ± 1 mm) and height (H) (using a Blumleiss altimeter) were measured.

To determine the amount of biomass in saplings (1 cm ≤ DBH < 10 cm) at the time of measurement in 2012, we established a subplot (25 m × 25 m) in the centre of each permanent plot. In each subplot, all saplings were identified at the species level and numbered. Across these six subplots, a total of 67 sapling species were found, of which 61 species were also found as adults. Of each individual, DBH and H (using a Blumleiss altimeter and pole altimeter for tree’s height < 5 m accuracy of ± 0.1 m) was measured.

To determine WD (g cm^−3^) and carbon content for each species, two wood core samples with a length of around 15 cm and a diameter of 0.5 cm were taken from opposite positions on the stem at DBH of one (with a DBH close to the mean DBH of that species in the plots) or more trees (see Nam et al. 2016). WD of each sample was determined as dry mass divided by its fresh volume (Chave 2005):

1 $${\text{WD }}=\frac{M}{{\left( {\frac{\pi }{4}} \right) \times {d^2} \times L}}$$

where *L* was the total length (cm), *d* the mean diameter of the sample (cm) and *M* the dry mass (g) of the sample (after oven-drying at 90 °C to constant mass). The WD of the species was calculated as the average of WD of the two wood core samples. In total, WDs of 97 species were determined at the Wood Science Laboratory of the Forestry University of Vietnam. The number of trees sampled for WD depended on their abundance: three trees for the five most abundance species, two for the subsequent 16 species and one for the rest. For the eight other species of which we could not determine WD (i.e. broken samples), we used the average WD of the species of the same genus if present, and otherwise the mean WD of species of the six plots which is 0.6 g cm^−3^ (Hertel et al. 2009). The two latter groups of species were excluded from the demographic analyses and were only included in estimates of forest mass. Carbon content in stem tissue was determined on subsamples that were finely ground at the Laboratory of the Ecology and Biodiversity Group of Utrecht University, The Netherlands (hereafter, Utrecht lab) using a CHN-Elemental Analyzer (CE instrument, inter-science BV, Breda, The Netherlands). In total, we determined the wood carbon content for 90 species (for all of which we had determined WD). In the case of the remaining 15 species, measurements failed due to defective samples. The species for which we were unable to determine WD and C content, were generally rare with only one or a few small stems in our plots.

Biomass in shrubs (including seedlings with stem DBH < 1 cm), was measured in 2012, by laying out four square frames (2 m × 2 m) in the four corners of each permanent plot. We then harvested all aboveground parts pertaining to shrubs and determined their fresh weight (FW). The dry weight (DW) and fresh weight (FW) ratio (DW/FW hereafter) was determined on subsamples of about 50 g FW, which were oven-dried at the Highland FRC at 75 °C until constant mass, and the total DW of shrubs was then calculated by multiplying the FW of the whole frame by the DW/FW ratio determined on the subsample. The carbon content in shrubs was determined on subsamples at the Utrecht Lab with the same methods as described above, however, without distinguishing between species.

Of standing dead trees (stumps included) we measured DBH and H in the same way as for the living trees. Trees were determined to be dead based on their shape and bark structure (in addition to lack of leaves) by us.

Biomass and carbon content of woody debris (including stems, branches and snags) and litter on the forest floor were determined within the same four square frames (2 m × 2 m each) using the same procedure in which shrubs were sampled.

### Measurements on belowground components

Soil carbon content was determined in two square soil profiles (50 cm × 50 cm × 100 cm depth, each) at two opposite corners within each permanent plot in 2012. These soil profiles were located at least 2 m away from the nearest standing trees to avoid hitting their roots. First, soil core samples (diameter of 4.5 cm and length of 20 cm) were taken at two depths (0–50 and 50–100 cm) and weighed to determine fresh mass, after which soil subsamples were dried to determine soil bulk density (g cm^−3^) at the Highland FRC. Second, the carbon content of these samples was determined as described for wood.

The amount of fine roots (diameter ≤ 2 mm) was determined in the abovementioned soil profiles, but we did not determine fine root content separately at each depth of the soil profiles. First, roots in soil were collected carefully by hand and cleaned under running water, and their FW was measured with an electronic balance (accuracy of ± 1 g). Second, ~ 50 g subsamples were collected and oven dried at 75 °C until constant mass and the DW/FW ratio was calculated. The carbon content in fine roots was determined as described for wood.

### Calculations

Biomass and organic matter components: Above ground biomass (AGB, kg tree^−1^) and coarse root (diameter > 2 mm) biomass (RB, kg tree^−1^) of each standing woody living tree (DBH ≥ 10 cm) in each measurement year were estimated using allometric equations that were developed for our study site (Nam et al. 2016):

2 $$AGB={\text{exp}}(-{\text{3}}.0{\text{51}}+0.{\text{966}}\;{\text{ln}}(DB{H^2}H)+0.{\text{3}}0{\text{5}}\;{\text{ln}}(WD))$$

3 $$RB={\text{exp}}(-{\text{1}}.{\text{651}}+{\text{1}}.{\text{934}}\;{\text{ln}}(DBH)+{\text{1}}.0{\text{6}}\;{\text{ln}}(WD))$$

with DBH and H the diameter at breast height (cm) and height (m) of each tree in each plot, AGB and RB are in kg dry biomass per tree. Equations were calibrated using destructive samples of 300 for AGB and 40 trees for RB (see Nam et al. 2016). For WD (g cm^−3^), we used the average value per species determined as described above.

We also used Eqs. (2) and (3) to determine the AGB and RB of saplings (1 cm ≤ DBH < 10 cm) and dead standing trees. To correct for the fact that part of the mass in dead trees is already decomposed, we used a correction factor based on visual assessment of decomposition state: 1 for dead trees with no signs of decomposition, 0.75 for moderately decomposed trees and 0.5 for highly decomposed trees (Latte et al. 2013).

The carbon content of each tree was estimated by multiplying the species specific C content by the AGB and RB values of each tree. As carbon content was determined on stem samples only, this assumes that C content of leaves and roots were similar to those of stems. For the 15 species (DBH ≥ 10 cm) and 6 species (1 cm ≤ DBH < 10 cm) for which we could not determine C content, we used the carbon content value of a species of the same genus if present, or otherwise the mean value (46.2%) of the carbon content of the 90 species for which we were able to determine carbon content.

The AGB and RB of each tree, total tree biomass (AGB and RB) and their respective carbon stocks (Mg ha^−1^) were then summed to determine total biomass and total carbon stock for standing living trees in each plot.

Amounts of carbon per ha in shrub, litter, woody debris and fine roots were estimated by multiplying the carbon content in each component by its dry mass. The carbon in soil organic matter (SOC, kg m^−3^) in each soil layer (0–50 cm and 50–100 cm) was estimated by multiplying the percentage of carbon in the soil (Ps, %), the soil bulk density (Sd, kg m^−3^) and the volume of each soil layer (V, m^3^) (Djomo et al. 2011; Ngo et al. 2013; Usuga et al. 2010). SOC in each soil profile (kg m^−3^) was the sum of the SOC in these two soil layers:

4 $${\text{SOC }}={\text{ Sd}} \times {\text{Ps}} \times {\text{V}}$$

Total carbon stock in each plot was the sum of the values of each component: standing living trees (DBH ≥ 10 cm), saplings (1 cm ≤ DBH < 10 cm), shrub, litter, woody debris, fine roots and soil.

Demographic rates: Demographic rates were calculated primarily for each species (or at the individual level) using trees with DBH ≥ 10 cm based on the measurements in 2004 and 2012. The rate expressed the demographic rates (growth, recruitment and mortality) for each species on a per capita basic. For all calculations of demographic rates and population level biomass dynamics we only considered the 42 most abundant species which had more than 20 individuals per species in the six permanent plots and their DBH was ≥ 10 cm. WD of all these species had been measured by us. These species accounted for more than 89% of total biomass and 87% of individuals of the total in the forest plots. In this analysis, the species *Dipterocarpus alatus* (total 56 individuals) was excluded due to a silvicultural treatment for this species after the first logging event (around 20 years ago) in one of the late-recovery forest plots (i.e. a number of small trees had been planted in gaps after logging), thus its estimated demographic rates do not reflect their natural values. Information about the study species can be found in the supporting information (see Table 1, S1).

The mortality rate per year (m, % year^−1^) between two measurements for each species was estimated as follows:

5 $${\text{m}}={\text{1}}00 \times \left( {\frac{{Ns1 - Ns2}}{{Ns1}}} \right) \times {{\text{t}}^{-{\text{1}}}}$$

where Ns_1_ is the number of living trees at measurement 1, Ns_2_ (N_S1_ ≥ N_S2_) is the number of surviving trees at measurement 2 and t is the time (in years) between the two measurement (Sheil et al. 2000; Wright et al. 2010). In this case rates were calculated primarily over the whole 2004–2012 period and thus t = 8 years.

Tree recruitment rate per year (r, % year^−1^) between two measurements were estimated as:

6 $${\text{r}}={\text{1}}00 \times \left( {\frac{{R12}}{{Ns1}}} \right) \times {{\text{t}}^{-{\text{1}}}}$$

where R_12_ is the number of new living trees at measurement 2, which recruited to DBH ≥ 10 cm.

The average growth rate (G_ind_) in terms of both DBH, AGB and TB (total biomass, AGB and RB) per tree per species were calculated following King et al. (2006) as:

7 $${\text{X}} - {{\text{G}}_{{\text{ind}}}}=\frac{1}{{Ns2}}\mathop \sum \limits_{{i=1}}^{{Ns2}} ({X_{2,i}} - {X_{1,i}})/t$$

where X refers to the entity (DBH, AGB or TB), suffixes 1 and 2 again refer to the two subsequent inventories and i denotes tree i (i = 1 → N_S2_). To make our calculations comparable with data from the literature, and because our estimate root mass was less accurate than those of AGB, biomass dynamics per tree are expressed in terms of AGB (AGB-G_ind_). Average relative growth rates (i.e. AGB growth per unit AGB or DBH growth per unit DBH, AGB-RGR and DBH-RGR, hereafter) were calculated by replacing X by ln(X) in Eq. (7).

Subsequently we calculated how different species contributed to biomass dynamics at the population level. Here population is defined as biomass dynamics of all individuals of a species with DBH ≥ 10 cm in a given area (i.e. hectare land area) which excludes smaller individuals (they constitute less than 5% of AGB). To this end, we calculated the net AGB increment per species per hectare (AGB-I_pop_, hereafter) as:

8 $${\text{AGB}} - {{\text{I}}_{{\text{pop}}}}={\text{AGB}} - {{\text{G}}_{{\text{pop}}}}+{\text{AGB}} - {{\text{R}}_{{\text{pop}}}}-{\text{AGB}} - {{\text{M}}_{{\text{pop}}}}$$

where AGB-G_pop_, AGB-R_pop_ and AGB-M_pop_ are the changes in biomass per species per hectare associated with growth, recruitment and mortality (i.e. thus expressed at population level), respectively. AGB-G_pop_was calculated as the total cumulated AGB growth of all surviving trees of a given species at the measurement 2 (essentially the same as multiplying G_ind_ for AGB from Eq. (7) by N_S2_). AGB-R_pop_was calculated as the total biomass of all newly recruited trees into the size class DBH ≥ 10 cm and AGB-M_pop_ as the total mass of trees present at the first census and dead at the second one. All these values were divided by plot area to normalize to per ha rates (Astrup et al. 2008).

### Statistical analysis

The data in the six plots were pooled. The differences in AGB (Mg ha^−1^) for each DBH size class between two measurements (2004 and 2012) of the six plots were determined by paired-test. Regression analyses were used to analyse the relationship between mean values per species for growth, recruitment, mortality and AGB-I_pop_ (all taken as dependent variables) and WD (as independent).

We also conducted a multiple regression with AGB-G_ind_ as dependent and WD and trees biomass (AGB size, average AGB per tree per species at the second census) as independents. Similarly, we did a multiple regression of population-level AGB growth (AGB-G_pop_) and net AGB increment rates (AGB-I_pop_) against WD and species abundance (number of individuals per species present in 2004, Ns1).

All calculations were performed by IBM SPSS statistics 21.0.

## Results

### Biomass and carbon stock in the forest

In the final census in 2012, the average total carbon stock in the forest was 355.4 ± 9.8 Mg C ha^−1^ (Table 1) ranging from 324 to 393 Mg C ha^−1^ across the six plots, to which biomass (AGB and RB) of living trees (DBH ≥ 10 cm) and soil carbon contributed approximately equally: 48.5 and 47.1%, respectively.

**Table 1:** Total carbon and biomass stocks (mean ± standard error of mean) in different components of the six plots in 2012

Component	Total	Standing woody trees		Fine roots	Shrub	Standing dead trees	Woody debris	Litter	Soil (0–100 cm)
		DBH (≥ 10 cm)	DBH (< 10)						
AGB (Mg ha^−1^)		327.2 ± 19.7	7.1 ± 0.6			6.36 ± 1.0			
RB (Mg ha^−1^)		43.4 ± 2.7	2.7 ± 0.2			1.06 ± 0.1			
Total mass (Mg ha^−1^)	406.6 ± 22.6	370.6 ± 22.5 (91.2%)	9.9 ± 0.8 (2.4%)	9.14 ± 0.5 (2.3%)	1.8 ± 0.1 (0.4%)	7.42 ± 1.1 (1.8%)	5.5 ± 0.9 (1.4%)	1.9 ± 0.1 (0.5%)	
Total carbon (Mg C ha^−1^)	355.4 ± 9.8	172.2 ± 10.4 (48.5%)	4.5 ± 0.3 (1.3%)	4.0 ± 0.2 (1.1%)	0.7 ± 0.1 (0.2%)	3.5 ± 0.5 (1.0%)	2.4 ± 0.4 (0.6%)	0.7 ± 0.1 (0.2%)	167.2 ± 3.5 (47.1%)

The average AGB of the large trees (DBH ≥ 10 cm) and saplings (DBH < 10 cm) accounted for 82.2% of the total forest biomass, while the coarse and fine root biomasses accounted for 13.5%. The other components, such as standing dead trees, woody debris, shrub and litter contributed very little to the total (4.3%). In terms of carbon stocks, the aboveground components (162.3 ± 9.0 Mg C ha^−1^) contributed less than the belowground components (193.1 ± 3.2 Mg C ha^−1^). Carbon in soil organic matter accounted for approximately 87% of the total amount of carbon in the belowground component, while roots (coarse and fine roots of both standing living and dead trees) accounted for 13%.

### AGB dynamics (2004–2012)

The total AGB of trees (DBH ≥ 10 cm) in each of the diameter size classes (classes: 10.0–29.9 and 30.0–49.9 cm) was higher in 2012 than in 2004 (*P* < 0.05), but for the larger class (DBH ≥ 50.0 cm) the difference was not significant (*P* > 0.05). In both censuses, the largest amount of AGB was found in trees of DBH class of 40 cm (range from 30 to 50 cm) (Fig. 1a). The proportion of the AGB (the percentage between AGB of this size class and the total AGB) for this DBH class (30–50 cm) did not change significantly over 8 years: it constitutes slightly over 30% of the total AGB. The number of trees within the DBH class of 10–30 cm, accounted for almost 75% of all trees, while their AGB was 20% of the total at both censuses (Fig. 1b).

Among species there were some changes in their ranking in total AGB (i.e., AGB of all individuals of a species) between 2004 and 2012, but the ten species with the highest AGB remained the same and accounted for 52% of total AGB in 2012. At both censuses the three dominant families, *Mangnoliaceae, Caesalpinioidae* and *Myrtaceae*, contributed about 40% to the total AGB.

We calculated biomass and tree dynamics over a period of 8 years (Table 2). The average net AGB increment in the six plots was 6.47 Mg ha^−1^ year^−1^, while AGB loss rate due to trees mortality was 2.50 Mg ha^−1^ year^−1^. AGB increase due to tree recruitment into the DBH ≥ 10 cm was very small (25% of AGB mortality rate). We found the average mortality rate to be lower than the recruitment rate across the 6 plots (*P* < 0.01).

**Table 2:** Biomass and tree dynamics (mean ± standard error of mean) of trees (DBH ≥ 10 cm) in the six plots in 8 years (2004–2012)

Demography	DBH increment rate (cm year^−1^)	AGB growth rate (Mg ha^−1^ year ^−1^)	AGB recruitment rate (Mg ha^−1^ year^−1^)	AGB mortality rate (Mg ha^−1^ year^−1^)	Net AGB increment rate (Mg ha^−1^ year^−1^)^a^	Mortality rate (% year^−1^)	Recruitment rate (% year^−1^)
Mean	0.35 ± 0.01	8.30 ± 0.32	0.67 ± 0.04	2.50 ± 0.27	6.47 ± 0.37	1.40 ± 0.10	2.54 ± 0.22

^a^Net AGB increment= (AGB growth + AGB of recruited trees − AGB lost by trees mortality)/8

### Individual level demographic rates and relation with wood density

**Fig. 2:**
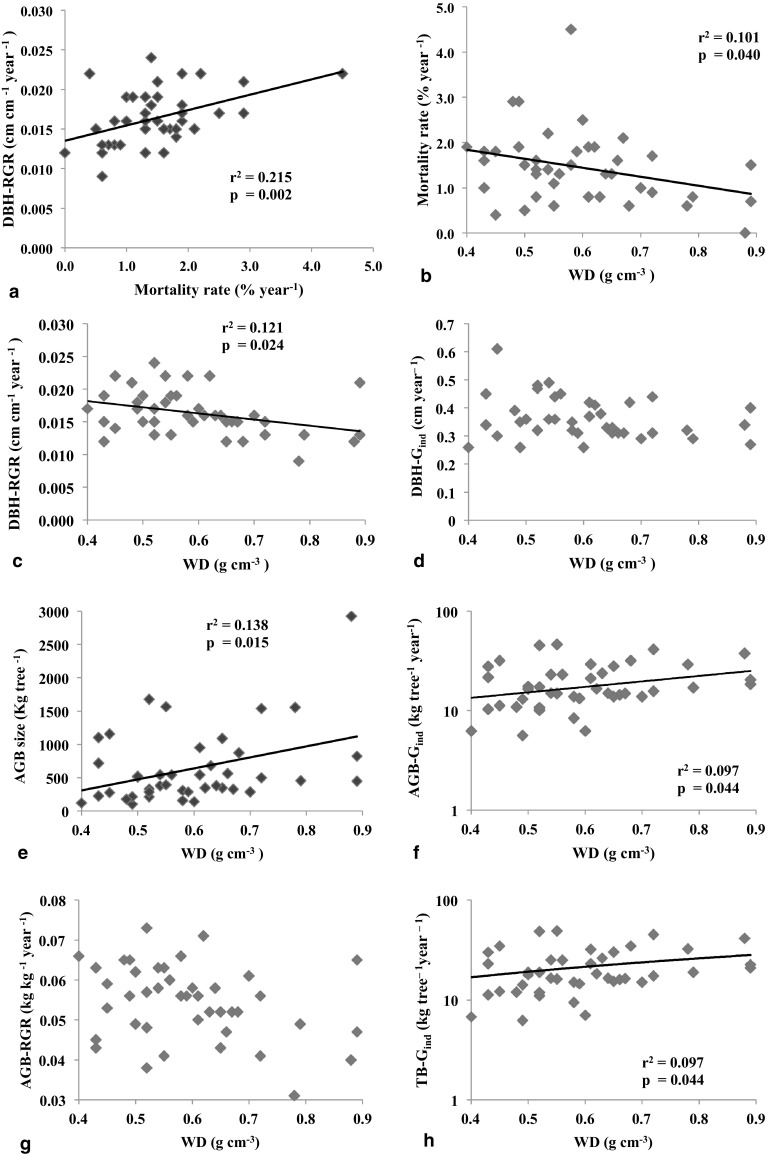
The relationships between species demographic rates and WD (g cm^−3^). **a** DBH-RGR and mortality rate, **b** mortality rate, **c** DBH-RGR, **d** DBH-G_ind_, **e** AGB size, **f** AGB-G_ind_, **g** AGB-RGR and **h** ln(TB-G_ind_). In **f, h**, Y axis has been log-transformed to present. Symbols indicate mean species values. The line indicates linear regression and is only shown when significant

When comparing between species there was a positive correlation (*P* < 0.01) between mortality rate and DBH-RGR (Fig. 2a). In contrast, mortality rate and species mean WD showed a weak negative relationship (*P* < 0.05, Fig. 2b). Similarly, we found a negative relationship between the DBH-RGR and WD (*P* < 0.05, Fig. 2c), whereas we did not find a significant relationship between DBH-G_ind_ and WD (*P* > 0.05, Fig. 2d). Contrary to the results for DBH, there was a positive relationship between AGB size (*P* < 0.05), AGB-G_ind_ (*P* < 0.05), TB-G_ind_ (*P* < 0.05), and WD (Fig. 2e, f, h). We did not find a relationship between AGB-RGR and WD (Fig. 2g). In the multiple regression of AGB-G_ind_ versus AGB size and WD, we found a strong positive relationship between AGB-G_ind_ and AGB size but the WD effect was no longer significant (Table 3). In short, species with high WD had relatively low relative diameter growth rates but were also relatively large and had comparatively high absolute biomass growth rates. These higher growth rates were not directly due to WD but rather resulted from the fact that high WD species were larger, and thus grew faster in absolute terms.

**Table 3:** Results of multiple-regression analysis across species: (i) at individual-level: mean aboveground biomass growth AGB-G_ind_ and WD and mean tree aboveground biomass (AGB size); and (ii) at population-level: mean aboveground biomass growth (AGB-G_pop_) and increment (AGB-I_pop_) versus WD and abundance (Ns1)

Variables	β	Sem	P
(i) Individual-level (AGB-G_ind_)			
WD	3.550	7.688	0.647
AGB size	0.014	0.002	0.0001
(ii) Population-level (AGB-G_pop_)			
WD	182.7	162.6	0.268
Abundance (Ns1)	2.635	0.561	0.0001
(iii) Population-level (AGB-I_pop_)			
WD	304.9	139.6	0.035
Abundance (Ns1)	1.718	0.481	0.001

### The relationship between wood density and AGB growth and net-AGB increment at population level

**Fig. 3:**
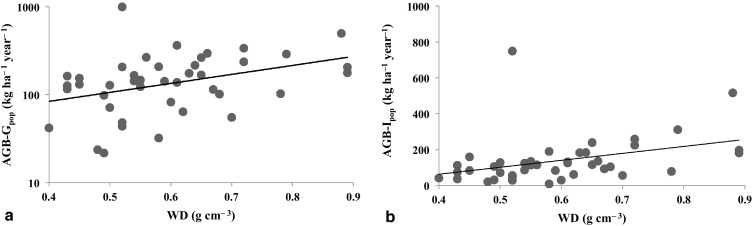
The relationship between **a** AGB-G_pop_ (kg ha^−1^ year^−1^) and WD (Y axis has been log-transformed to present), **b** AGB-I_pop_ (kg ha^−1^ year^−1^) and WD of the 42 most abundant species

Among the 42 most abundant species we found a positive relationship between WD and both its population level AGB-G_pop_ (*P* < 0.05), and AGB-I_pop_ (*P* < 0.05) (Fig. 3a, b). Multiple regression showed that abundance (Ns1) was significantly related with AGB-G_pop_ and AGB-I_pop_, but the relation with WD was only significant for AGB-I_pop_ and insignificant to AGB-G_pop_ (Table 3). Nevertheless, the most abundant species (*Paramichelia braianensis*), with a relatively low WD (0.52 g cm^−3^), had the largest contribution to these measures with AGB-G_pop_ of 0.98 Mg ha^−1^ year^−1^ and AGB-I_pop_ of 0.75 Mg ha^−1^ year^−1^ (Fig. 3).

## Discussion

### Biomass and carbon stock in the forest

To characterize our forest relative to others we first briefly discuss standing biomass and carbon stocks. The total carbon stock at our site was 355.4 Mg C ha^−1^ with 42% of this (151.0 Mg C ha^−1^) in aboveground biomass (AGB) of living trees. This is within the range of values found for mature tropical forests 57–375 Mg C ha^−1^ across the tropics (Lewis et al. 2013; Niiyama et al. 2010). It should be noted that the forest plots analysed by us had been selectively logged about 30 years before our measurements. As we have no data on the pre-logging biomass it is difficult to determine whether AGB levels had already recovered to the pre-logging state. The only long-term study that we know of on post-logging biomass dynamics (Gourlet-Fleury et al. 2013) showed that 24 years after logging in African semi-deciduous forest, AGB levels had more than recovered to their pre-logging state.

The carbon stock in the soil was 167 Mg C ha^−1^ accounting for almost half of the total carbon stock in the forest. Together with the amount of C in roots, which we estimated to be 24 Mg C ha^−1^, this means that about 54% of forest carbon was found below ground. Estimates of soil carbon vary widely between studies, and belowground fractions of 33–66% have been reported (Djomo et al. 2011; Gibbs et al. 2007; Malhi et al. 2009).

Part of the variation in the estimates of soil C is associated with the variation in sampling depth. Various studies estimate soil carbon to a depth of 30 cm (e.g. Djomo et al. 2011; Wei et al. 2014). Here we measured soil C down to 100 cm and found that 2/3 (116.1 Mg C ha^−1^) of C was in the top 0–50 cm and 1/3 (51.2 Mg C ha^−1^) in the deeper layer (50–100 cm). This suggests that limiting measurements to the top 30 cm would have led to a serious underestimation of soil C. In addition, a recent study (Ngo et al. 2013) sampled soil C down to 300 cm depth and found that 40% of C was located deeper than 1.0 m. Thus, our work and that of others point to the importance of measuring soil C well beyond 1.0 m depth.

### Biomass dynamics

In this study we assessed forest level carbon and biomass dynamics, and biomass dynamics of different species at the individual and population level in a forest that was recovering for 30 years from a significant logging event. Particularly we addressed the extent to which species biomass dynamics are associated with species WD. At the forest level, net aboveground biomass increment rates were rather high suggesting that 30 years post-logging the forest is still in a recovery stage. Among species, we found negative relationships of mortality rate with diameter growth rates and species wood density. We also found that high WD species tended to be larger in terms of standing biomass and thus exhibited higher rates of biomass growth and net above ground biomass increment both at the individual and population level. These findings support the view that high WD species contribute more to biomass and biomass increment than low wood density species in tropical forest.

In our study the net increment of aboveground biomass at forest level was high (6.47 Mg ha^−1^ year^−1^) compared to values generally reported for mature tropical forest (from ^−1^.0 to 4.8 Mg ha^−1^ year^−1^) (e.g. Chave et al. 2008a; Gourlet-Fleury et al. 2013). Our census spanned the period between 23 and 31 years after logging. Gourlet-Fleury et al. (2013) analysed forests that had been selectively logged between 1984 and 1987 and found that the mean net AGB increment over the subsequent 24 years ranged between 4.8 and 8.0 Mg ha^−1^ year^−1^, the range encompasses the value reported by us, but was 2–4 times higher than the net AGB increment value they found in nearby plots of undisturbed forests. Both our results and those of Gourlet-Fleury et al. (2013) suggest that forests may exhibit accelerated rates of biomass increment for several decades following a selective logging event. However another study (Rutishauser et al. 2016) reported 2–13% reduction in tree growth over 25 years after a selective logging event, indicating that these effects can vary between sites.

Net AGB increment is roughly the difference between AGB growth (the combined growth of all surviving trees in a stand) and AGB losses through mortality (i.e., biomass gains through recruitment were very small). Thus, high biomass increment rates could be the result of acceleration of tree growth, suppression of mortality, or both. Both processes may have played a role at our site. AGB growth rate was found to be 8.3 Mg ha^−1^ year^−1^ which is within the range of values reported for mature tropical Amazonian forests (Malhi et al. 2011), but higher than most reported values (e.g. Djomo et al. 2011; Hertel et al. 2009). Losses in biomass as a result of tree mortality were indeed lower than the range of values found in Amazonian forests in the review of Malhi et al. (2009).

How could relative fast growth and low mortality several decades after logging be explained? At our site the disturbance by logging was most likely severe; 30–35% of the standing volume was probably directly harvested with a further 10–15% of trees being killed during, or shortly after, logging (Con et al. 2007; MARD 2005; Sam et al. 2007). Such disturbances open up the forest at least initially, and increase light availability, which stimulates growth particularly that of light-demanding species such as long-lived pioneers (Gourlet-Fleury et al. 2013; Pena-Claros et al. 2008; Sist and Nguyen-Thé 2002). While direct effects of opening the forest most probably faded out in our case, the higher light intensity may have enabled fast-growing individuals to reach the canopy, which could have increased their survival chance in subsequent years. Evidently these effects may vary and likely depend on environmental factors such as soil fertility and species composition (i.e., the presence of light demanding species).

### Relationship between wood density and growth

In the second part of this paper, we analysed the extent to which interspecific variation in demographic and associated biomass dynamics were associated with species WD. We found a negative correlation between DBH-RGR and WD. This result is consistent with other findings (Iida et al. 2012; King et al. 2006; Poorter et al. 2010) and with theoretical models showing that for vertical growth it is mechanically more efficient to produce thicker stems with low WD than thinner stems with high WD (Anten and Schieving 2010).

The general notion is that low WD is also associated with rapid growth in terms of biomass. The arguments are that less dense woody tissue permits higher hydraulic conductivity, therefore greater photosynthetic capacity of trees than higher WD (Chave et al. 2009; Keeling et al. 2008). Furthermore, a low WD might be part of a suite of traits (high SLA, high leaf photosynthetic capacity, etc.) associated with rapid growth and a high WD with an opposite set of traits (Chave et al. 2009). Few studies however have considered the relationship between WD and AGB increment in a natural forest and none that we know of considered the relationship between TB growth and WD. Interestingly and contrary to the general view, we found a positive relation between average annual AGB and TB growth, and WD at individual level. Our results for AGB are consistent with the findings of Keeling et al. (2008).

To determine WD, we sampled 1–3 individuals per species with a DBH close to the species mean in our plots. WD can differ between individuals of the same species among other things as a function of DBH (e.g. Nock et al. 2009; Woodcock and Shier 2002). Differences in WD observed in our study could in theory really have reflected differences in DBH. In our study however differences in DBH were small and not significantly correlated with WD, and we are therefore confident that this issue did not significantly bias the results of our study.

Keeling et al. (2008) proposed several factors that could explain a positive WD growth relationship. These include high WD tending to have: (1) high leaf and branch longevity (e.g. Kitajima and Poorter 2010) resulting in larger and deeper crowns (Keeling et al. 2008) and thus higher whole-plant photosynthesis (Aiba and Kohyama 1997; Lusk 2002), (2) lower respiration rates (Reich et al. 2006; Wright et al. 2004), and a larger resistance to pathogens or hydrological stress preventing (periods of) inhibited growth.

Our results however suggest a different explanation. There was a negative correlation between mortality rates and WD (*R*^2^ = 0.101) consistent with other studies (Chave et al. 2009; King et al. 2006) and a positive relationship between WD and AGB size. In multiple regressions of growth versus AGB size and WD, only AGB size was significant. Together these results indicate that individuals of high WD species have lower mortality chances and can on average grow to larger size, and larger trees in turn have higher absolute AGB growth rates.

Similar to the mean rates per tree we also found strong positive relationships between WD and both total AGB growth and total net AGB increment at the tree population level. Our findings support the results of Chave et al. (2008a), who found high WD species gained more biomass than low WD species in different forest sites in three continents (Africa, America and Asia).

Our results indicate that on average a given species with a high WD contributes more to the net biomass increment in a forest than low WD species, albeit indirectly through its effect on size. Given that high WD probably also entails slower decomposition, this pattern might be strengthened if considered at the ecosystem level. This result should also be considered in relation to forest management. Selective logging is commonly used for commercial timber production, so if loggers tend to focus on species with relatively high WD they consequently also focus on those that have on average a relatively large contribution to biomass increment. Thus selective logging may disproportionally affect high WD species.

## Electronic supplementary material

Below is the link to the electronic supplementary material.

Supplementary material 1 (PDF 133 KB)
