# Supplementary material for: Biomass dynamics in a logged forest: the role of wood density
Source: J Plant Res. 2018 May 30;131(4):611–21. doi: 10.1007/s10265-018-1042-9 (PMC6015617; doi:10.1007/s10265-018-1042-9)
Supplement: Supplementary file 1 — Supplementary material 1 (PDF 133 KB) [file 10265_2018_1042_MOESM1_ESM.pdf]

Electric supplementary materials

Title:

Biomass dynamics in a logged forest: the role of wood density

Authors:

Vu Thanh Nam

Department of Biology, Utrecht University, Padualaan 8, 3584 CH Utrecht, the Netherlands

Present address: Vietnam Administration of Forestry, No 2, Ngoc Ha, Ba Dinh, Hanoi, Vietnam

Email: Nam@vnforest.gov.vn

Journal:

Journal of Plant Research

Corresponding author:

Niels P.R. Anten

Centre for Crop Systems Analysis, Wageningen University, Droevendaalsesteeg 1, 6708 PB Wageningen, the Netherlands

Email: niels.anten@wur.nl

Marijke van Kuijk

Department of Biology, Utrecht University, Padualaan 8, 3584 CH Utrecht, the Netherlands

Email: m.vankuijk@uu.nl

Table S 1: Characteristics of the study species

No	Species	WD	N2 in 2012 (trees 6 ha^{-1})	Mortality rate (% $year^{-1}$)	Recruitment rate (% $year^{-1}$)	DBH-G_{ind} rate (cm $year^{-1}$)	AGB-G_{ind} rate (Kg $tree^{-1}$ $year^{-1}$)	TB-G_{ind} rate (Kg $tree^{-1}$ $year^{-1}$)	AGB-G_{pop} rate (Kg ha^{-1} $year^{-1}$)	AGB-I_{pop} rate (Kg ha^{-1} $year^{-1}$)
1	*Paramichelia braianensis*	0.52	153	0.80	1.96	0.51	45.24	48.37	987.66	748.47
2	*Symplocos sumuntia*	0.49	149	1.93	4.57	0.26	5.65	6.24	97.90	105.41
3	*Syzygium cuminii*	0.66	151	1.60	2.39	0.31	14.35	15.93	294.23	135.41
4	*Syzygium zeylanicum*	0.65	140	1.28	2.56	0.32	13.86	15.35	263.36	239.50
5	*Machilus ordoratissima*	0.61	131	1.86	2.89	0.38	21.11	23.05	362.36	131.77
6	*Naphelium cuspidatum*	0.79	129	0.80	3.10	0.29	17.02	18.84	289.30	310.00
7	*Polyalthia corasoides*	0.58	108	1.47	2.21	0.32	13.83	15.09	207.51	188.50
8	*Syzygium wightianum*	0.72	102	1.79	1.43	0.31	15.77	17.44	236.62	224.49
9	*Naphelium melliferum*	0.64	101	1.30	1.95	0.33	14.99	16.47	214.80	184.71
10	*Dialium cochinchinensis*	0.88	95	0.0	2.53	0.36	37.66	41.40	495.82	515.62
11	*Lipthocarpus ducampii*	0.89	92	1.48	4.11	0.41	18.38	20.89	205.28	197.38
12	*Ormosia balansae*	0.52	91	1.39	2.93	0.48	17.23	18.89	206.71	55.68
13	*Aglai elaeagnoidea*	0.59	89	1.83	4.17	0.31	13.26	14.53	141.39	82.72
14	*Gironniera subaequalis*	0.43	86	1.03	3.25	0.34	10.38	11.25	115.89	111.23
15	*Wendlandia paliculata*	0.60	85	2.53	0.76	0.26	6.26	7.01	82.43	29.17
16	*Microcos paniculata*	0.54	84	1.35	3.04	0.37	15.03	16.42	165.34	123.47
17	*Morus alba*	0.45	82	1.83	1.83	0.30	11.22	12.15	130.89	82.76
18	*Lipthocarpus vestitus*	0.56	79	1.30	1.62	0.45	23.02	25.01	264.70	114.43
19	*Sinosideroxlon Bonii.*	0.89	60	0.68	1.82	0.28	20.40	22.55	176.81	181.85

20	*Antidesma ghasembilla*	0.67	59	2.05	2.95	0.31	14.88	16.39	114.04	91.90
21	*Cinnamomum bejolghota*	0.50	59	1.50	3.75	0.37	17.41	18.83	127.67	127.99
22	*Garuga pierrei*	0.63	57	0.80	3.46	0.38	23.93	26.25	175.48	183.63
23	*Castanopsis indica*	0.72	56	0.94	1.65	0.45	41.34	45.08	337.64	258.55
24	*Symplocos conchinchinensis*	0.40	53	1.86	3.46	0.27	6.25	6.77	41.66	42.58
25	*Cinnamomum ovantum*	0.55	52	1.14	0.45	0.36	14.83	16.23	123.60	112.98
26	*Prumus arborea*	0.54	48	2.22	3.06	0.37	23.15	25.20	142.75	85.38
27	*Aglai spectabilis*	0.65	38	1.25	0.63	0.34	27.93	30.16	167.56	116.34
28	*Alstonia scholaris*	0.43	38	1.83	0.91	0.50	27.68	29.97	161.44	35.64
29	*Schefflera heptaphylla*	0.43	37	1.56	0.63	0.34	21.66	23.04	126.36	74.20
30	*Endospermum chinensis*	0.45	35	0.42	2.50	0.61	31.93	34.65	154.30	159.44
31	*Elaeocarpus griffithii*	0.62	32	1.85	4.17	0.41	16.68	18.32	63.94	62.19
32	*Cratoxylum sp*	0.52	32	1.29	2.59	0.32	10.07	11.03	43.63	45.65
33	*Vitex trifolia*	0.52	30	1.61	1.21	0.34	10.72	11.85	48.25	27.69
34	*Syzygium zeylanicum*	0.58	30	4.51	2.43	0.35	8.38	9.46	32.12	8.38
35	*Artocarpus nitidus*	0.70	29	0.96	2.40	0.29	13.79	15.15	55.17	55.67
36	*Caranium album*	0.61	29	0.83	0.42	0.45	29.42	31.95	137.31	125.89
37	*Croton argyrata*	0.50	28	0.46	0.93	0.35	31.89	34.66	71.27	71.98
38	*Engelhardtia roxburghiana*	0.68	22	0.63	1.88	0.42	46.20	49.09	101.00	103.50
39	*Michelia mediocris*	0.55	22	0.63	1.88	0.46	29.23	32.39	146.30	135.02
40	*Craibiodendron scleranthum*	0.78	22	0.57	0.57	0.32	13.02	14.18	102.30	78.27
41	*Symplocos laurina*	0.49	20	2.88	9.62	0.35	10.89	11.99	21.71	31.91
42	*Knema pierei*	0.48	20	2.94	5.15	0.39	16.45	17.86	23.60	20.87

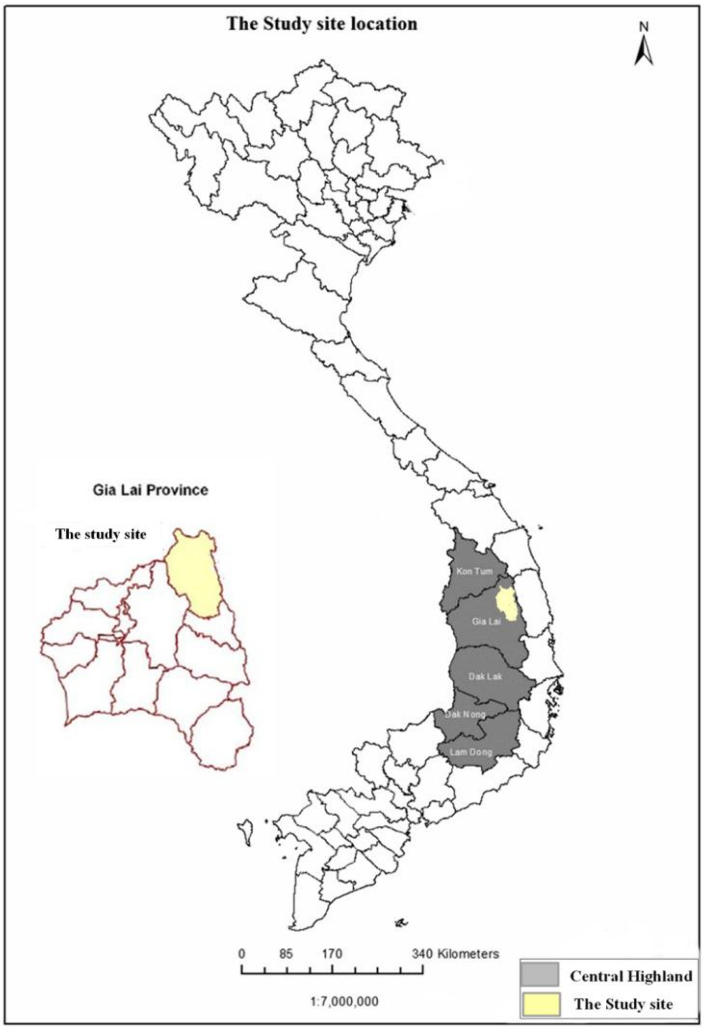

Fig. S1: Map of the study site.
